# Non-Invasive Measurement of Hemodynamic Parameters via Whole-Body Impedance Cardiography Among Hospitalized Heart Failure Patients: An Effective Alternative to Invasive Right Heart Catheterization?

**DOI:** 10.3390/jcdd12040128

**Published:** 2025-04-02

**Authors:** Felix Ausbuettel, Sabah Khwamurad, Murad Haj Abdo, Sebastian Kerber, Karin Nentwich, Martina Hautmann, Sebastian Barth

**Affiliations:** 1 Department of Cardiology, University Hospital Marburg, Baldingerstraße, 35043 Marburg, Germany; 2 Department of Cardiology, Cardiovascular Center Bad Neustadt/Saale, Von-Guttenberg-Straße 11, 97616 Bad Neustadt/Saale, Germany

**Keywords:** cardiac output, right heart catheterization, impedance cardiography

## Abstract

(1) Background: The measurement of hemodynamic parameters has proven to be crucial in the treatment of hospitalized heart failure patients, necessitating invasive measurement by right heart catheterization (RHC). The reliability of whole-body impedance cardiography (ICG) among this cohort has not been investigated to date; (2) Methods: The RHC and whole-body ICG examinations measured cardiac output (CO), the cardiac index (CI), total peripheral resistance (TPR), and pulmonary vascular resistance (PVR). To assess the accuracy of the whole-body ICG measurement, bias and precision were calculated as the mean difference and the twofold standard deviation between the average values of measurements; (3) Results: A total of 203 patients were analyzed. No significant bias was observed between the non-invasive CO and CI measurements when compared with the RHC measurements (−0.14 ± 2.56 L/min, *p* = 0.1; −0.09 ± 1.3 L/min/m^2^, *p* = 0.06), but a significant bias occurred in the measurement of non-invasive TPR and non-invasive PVR (−1243 ± 3510 dyn × s^−1^ × cm^−5^, *p* = 0.001; −121 ± 504 dyn × s^−1^ × cm^−5^, *p* < 0.001); (4) Conclusions: CO and CI can be measured with whole-body ICG among hospitalized CHF patients with acceptable accuracy. The reliability of measuring TPR and PVR should be further investigated.

## 1. Introduction

Congestive heart failure (CHF) is a highly prevalent disease in Western industrialized countries that affects over 23 million people worldwide [1], and it has a considerable impact on morbidity and mortality [2,3]. Owing to ongoing demographic changes, a further increase in prevalence is expected in the coming decades [3].

Invasive right heart catheterization (RHC) remains the gold standard for measuring hemodynamic parameters among CHF patients, especially because cardiac output (CO) has been demonstrated to predict long-term outcomes [4]. Nevertheless, RHC has certain drawbacks due to its invasiveness. As a result, non-invasive measurement methods, such as whole-body impedance cardiography (ICG), have been developed to facilitate the accurate assessment and monitoring of hemodynamic parameters in these patients. The non-invasive cardiac system (NICaS^TM^) shows a correlation between non-invasively measured CO (NI-CO) and RHC-measured CO [5], resulting in FDA approval for further clinical use. While whole-body ICG measurement has a clear advantage over RHC due to its non-invasiveness, its practical applicability can still be hampered by patient-specific physiological fluctuations in hemodynamic parameters and comorbidities such as severe valvular heart disease.

Previous studies have focused primarily on the intraoperative monitoring of CO in an isolated collective of patients undergoing coronary artery bypass grafting (CABG) surgery, while the investigation of CHF patients not undergoing cardiac surgery has been largely neglected [6,7,8,9,10]. In addition to CO measurement, whole-body ICG allows the calculation of additional hemodynamic parameters, such as the cardiac index (CI) and total peripheral resistance (TPR), which may eliminate the need for invasive RHC for treating patients. Therefore, the present study investigated the reliability of whole-body ICG in measuring hemodynamic parameters compared with RHC in a broader “real-world” cohort that was hospitalized due to CHF.

## 2. Materials and Methods

In the present single-center study, all patients who underwent invasive RHC and non-invasive whole-body ICG measurement due to CHF between 2021 and 2023 were enrolled for further analysis. The exclusion criteria for whole-body ICG measurement were as follows: severe aortic valve regurgitation, severe mitral valve regurgitation, severe aortic valve stenosis, obesity with a body mass index of >40 kg/m^2^, residual severe peripheral edema, severe peripheral arterial occlusive disease, restlessness, dialysis, sepsis, and tachyarrhythmia absoluta due to atrial fibrillation at the time of measurement.

The RHC and whole-body ICG measurements were performed by two different operators who were blinded to the results of the opposite procedure. A Swan–Ganz catheter was advanced into the pulmonary artery via the vena femoralis for invasive RHC measurement, whereby the correct placement was verified by the respective pressure waveforms under fluoroscopic control. The CO was measured via the Fick method and indexed as CI on the basis of body surface area calculated via the Du Bois formula, in line with previous studies [11,12]. The pulmonary vascular resistance (PVR) was calculated accordingly using the following formula: PVR = 80 × (mean pulmonary arterial pressure [mPAP] − pulmonary capillary wedge pressure [PCWP])/pulmonary arterial flow [13]. The TPR was measured based on the following formula: mean arterial pressure (MAP)/CO [14].

Non-invasive measurements of hemodynamic data were obtained with whole-body ICG provided by the NICaS^TM^ system (NI-medical, Ra’anana, Israel). The measurement was performed before or after RHC, and the corresponding leads were placed between one wrist and the contralateral ankle. The recording duration of a single whole-body ICG measurement was automatically ended by the device after an interval of five minutes. In the case of insufficient measurements or absolute arrhythmia due to atrial fibrillation (AF), multiple measurements were performed. For the calculation of the non-invasive PVR (NI-PVR), the pulmonary artery systolic pressure (PASP) was measured via transthoracic echocardiography using the peak velocity of the continuous wave doppler across the tricuspid valve regurgitation. The mPAP was calculated in accordance with the previous literature [15] via the following formula: mPAP = PASP × 0.61 + 2 mmHg. For the calculation of the NI-PVR, a PCWP of 20 mmHg was assumed in all cases.

### Statistical Analysis

Categorical variables are reported as absolute and relative frequencies (%). Continuous normally distributed variables are presented as the mean and standard deviation (SD), and continuous non-normally distributed variables are presented as the median and interquartile range (IQR, 25th–75th percentile). The probability of a normal distribution was assessed via the Shapiro–Wilk test.

To assess the accuracy of whole-body ICG for the non-invasive measurement of hemodynamic parameters compared with invasive RHC, the respective mean values were compared via paired Student’s *t*-tests. Furthermore, the extent of the correlation was tested via linear regression analysis and the Pearson’s correlation coefficient, which was then illustrated in a linear regression plot. A bias of the whole-body ICG was defined as the mean difference between the RHC and whole-body ICG values. The precision of the whole-body ICG measurement was calculated as two SDs of the mean differences between the RHC and whole-body ICG values and is presented in a Bland–Altman plot, analogous to that in the previous literature [6]. A two-sided tested *p*-value of ≤0.05 was considered statistically significant.

All of the statistical analyses were conducted with R Studio V4.3.1 (R Foundation for Statistical Computing, Vienna, Austria) using the “dplyr”, “ggplot2”, and “BlandAltmanLeh” packages and GraphPad Prism 6.0 (Dotmatics, Boston, MA, USA). The graphical abstract was designed with BioRender.com (Science Suite Inc., Toronto, ON, Canada).

## 3. Results

### 3.1. Baseline Characteristics

A total of 203 patients were included for further analysis during the observation period. All patients were hospitalized due to new-onset or aggravated dyspnea. The study cohort was predominantly male, with 85.7% (174/203) exhibiting structural heart disease and 43.8% (89/203) presenting with CHF symptoms of New York Heart Association (NYHA) class ≥III. In terms of the left ventricular ejection fraction (LVEF), 53.7% (109/203) of patients presented with heart failure with preserved ejection fraction (HFpEF), 34% (69/203) of patients with heart failure with reduced ejection fraction (HFrEF), and 12.3% (25/203) with heart failure with mildly reduced ejection fraction (HFmrEF).

In line with the inclusion criteria, no patient had severe mitral valve regurgitation, severe aortic valve regurgitation, or severe aortic valve stenosis. However, 15.8% (32/203) of patients had high-grade tricuspid valve regurgitation. An overview of the clinical characteristics of the cohort is provided in Table 1, and the echocardiographic characteristics are listed in Table 2.

### 3.2. Comparison of Non-Invasive and Invasive Measurements of Hemodynamic Parameters

There was no significant difference between the mean NI-CO and RHC-CO (4.1 ± 1.3 L/min vs. 4 ± 1.2 L/min, *p* = 0.1), and there was no significant difference between the mean non-invasive CI (NI-CI) and RHC-CI (2.1 ± 0.6 L/min/m^2^ vs. 2.1 ± 0.6 L/min/m^2^, *p* = 0.06). In contrast, the mean TPR and PVR differed significantly between the two measurement methods, with non-invasive TPR (NI-TPR) and NI-PVR underestimating the RHC-TPR (2225 ± 3314 dyn × s^−1^ × cm^−5^ vs. 1733 ± 590 dyn × s^−1^ × cm^−5^, *p* = 0.001) and RHC-PVR (386 ± 218 dyn × s^−1^ × cm^−5^ vs. 220 ± 162 dyn × s^−1^ × cm^−5^, *p* < 0.001), respectively. A detailed overview of the hemodynamic parameters, as measured using whole-body ICG and RHC, is provided in Table 3.

**Figure 1:**
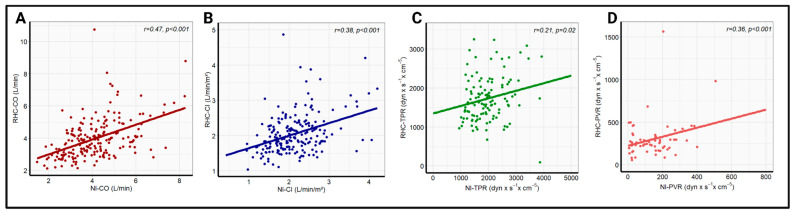
Linear correlation analysis between RHC-CO and NI-CO (**A**), RHC-CI and NI-CI (**B**), RHC-TPR and NI-TPR (**C**), and RHC-PVR and NI-PVR (**D**) in the overall cohort. NI-CI—non-invasively measured cardiac index, NI-CO—non-invasively measured cardiac output, NI-PVR—non-invasively measured pulmonary vascular resistance, NI-TPR—non-invasively measured total peripheral resistance, RHC-CI—right heart catheter-derived cardiac output, RHC-CO—right heart catheter-derived cardiac index, RHC-PVR—right heart catheter-derived pulmonary vascular resistance, RHC-TPR—right heart catheter-derived total peripheral resistance.

Pearson correlation analysis revealed a significant correlation between the whole-body ICG and RHC data for all four hemodynamic parameters. The strongest correlation coefficient was recorded for CO (r = 0.47, *p* < 0.001), followed by CI (r = 0.38, *p* < 0.001), and PVR (r = 0.36, *p* < 0.001). A significant, but comparatively poor, correlation was found for TPR (r = 0.21, *p* = 0.02). The linear regression analysis with the corresponding correlation analysis is illustrated in Figure 1. Additionally, there was a strong correlation between the echocardiographic and invasive measurement of PASP (r = 0.71, *p* < 0.001), which is presented in Figure S1. No significant change in correlation coefficients was observed after the exclusion of patients with high-grade tricuspid regurgitation, as shown in Figure S2.

**Figure 2:**
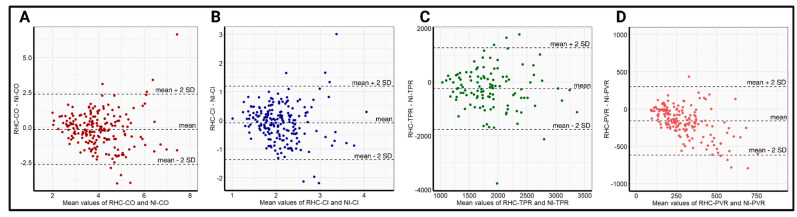
Bland–Altman plots comparing the precision (mean difference ± 2 SD) between RHC-CO and NI-CO (**A**), RHC-CI and NI-CI (**B**), RHC-TPR and NI-TPR (**C**), and RHC-PVR and NI-PVR (**D**) in the overall cohort. NI-CI—non-invasively measured cardiac index, NI-CO—non-invasively measured cardiac output, NI-PVR—non-invasively measured pulmonary vascular resistance, NI-TPR—non-invasively measured total peripheral resistance, RHC-CI—right heart catheter-derived cardiac output, RHC-CO—right heart catheter-derived cardiac index, RHC-PVR—right heart catheter-derived pulmonary vascular resistance, RHC-TPR—right heart catheter-derived total peripheral resistance, SD—standard deviation.

The relatively small differences in the mean values, in addition to the high correlations of NI-CO and NI-CI, with the respective RHC data, resulted in a bias and precision of −0.14 ± 2.56 L/min and −0.09 ± 1.3 L/min/m^2^, respectively, for the whole-body ICG. Owing to the significant differences in the obtained mean values of TPR and PVR with comparatively poorer correlation, the bias and precision in the respective TPR and PVR measurements were worse, at −1243 ± 3510 dyn × s^−1^ × cm^−5^ and −121 ± 504 dyn × s^−1^ × cm^−5^, respectively. Bland–Altman plots displaying the bias and precision of whole-body ICG compared with RHC in measuring hemodynamic parameters are provided in Figure 2.

Limited by the retrospective study design, 22.2% of patients (45/203) did not have a simultaneous whole-body ICG measurement on the day of RHC measurement. The maximum latency between the measurements in these patients was one day. Nevertheless, the accuracy of whole-body ICG measurement may have been affected compared to RHC measurements.

## 4. Discussion

The assessment of hemodynamic cardiac parameters remains crucial in patients suffering from CHF, but this particular collective has been largely neglected in previous studies regarding whole-body ICG measurement. The present study, therefore, investigated the hemodynamic parameters of hospitalized CHF patients with whole-body ICG via the NICaS^TM^ system and evaluated its accuracy via comparisons with data derived from invasive RHC. The present results indicated a fair correlation between NI-CO and RHC-CO, as well as between NI-CI and RHC-CI, while there were significant deviations between the TPR and PVR measurements.

In terms of clinical characteristics, the present cohort was older and had a greater prevalence of coronary artery disease (CAD), AF, and arterial hypertension compared to the patients in the ESC heart failure (ESC-HF) registry [16] and the global congestive heart failure (G-CHF) registry [17], which are two large multicenter registries investigating CHF patients. Additionally, the majority of the present cohort presented with HFpEF, whereas HFrEF constituted the most prevalent CHF phenotype in the current CHF registries [16,17].

The mean CO and CI were slightly lower in the present cohort, but there was close agreement between non-invasive and invasive measurements, which agreed with the previous findings, reporting a bias of whole-body ICG in measuring CO with a range of 0.001–1.62 L/min [5,7,9,10,18,19]. However, the correlation coefficients in the present study were lower than those reported in previous studies [5,6,20], even though the correlation with the invasive RHC data remained significant. Hereby, the influence of differences in clinical cohort characteristics on the correlation cannot be investigated, as most studies did not publish cohort characteristics. Imhof et al. investigated the correlation of NI-CO with RHC-CO among CABG patients, and they reported even lower correlation coefficients compared to the present study [18]. These differences could be explained by discrepancies between the population used for calibration of the whole-body ICG and this study cohort, as well as by alternating peripheral perfusion due to perioperative variations. Moreover, the influence of both factors on the results of the present cohort cannot be ruled out either.

Although the recorded NI-TPR and NI-PVR values significantly correlated with the RHC values, the precision of the NI-TPR and NI-PVR measurements was significantly worse than that of the CO and CI measurements. While there is a lack of data on the comparability of NI-TPR to RHC-TPR, NI-PVR has been investigated by Taniguchi et al. in patients with precapillary pulmonary hypertension [21]. Taniguchi et al. reported a sufficient correlation between NI-PVR and RHC-PVR, with the subsequent accurate detection of an increased PVR; however, these results could not be extrapolated to the present cohort, as it included patients with postcapillary pulmonary hypertension due to underlying CHF, resulting in normal values of PVR. The significantly lower precision of the NI-PVR could also have been reinforced by the hypothetical assumption of a PCWP value of 20 mmHg in the formula. In general, the PCWP value represents a crucial parameter in the calculation of NI-PVR, as its estimation through non-invasive methods tends to be complicated. Taniguchi et al., therefore, already applied a hypothetical average PCWP value of 12 mmHg, as they were investigating a collective of patients with precapillary pulmonary hypertension. The present collective consisted of patients hospitalized due to left-sided CHF, so, in principle, an increased PCWP value had to be expected. Even though this approach followed the same methodology used by Taniguchi et al., under the assumption of postcapillary pulmonary hypertension, residual uncertainty remains in the calculation. This uncertainty could be mitigated through additional echocardiographic calculations of PCWP, as recently described by Martelli et al. [22], which nevertheless was not possible in the present collective due to the retrospective study design. As the invasively measured average PCWP was actually measured at 14 ± 8 mmHg, it was slightly overestimated by the hypothesized PCWP value of 20 mmHg.

The echocardiographic measurement of PASP may be inaccurate, particularly in the case of high-grade tricuspid valve regurgitation. A negative influence of the echocardiographic measurement of PASP on the precision in the present collective was assessed as unlikely, due to the strong correlation with the invasive measurement of PASP.

An essential difference from previous studies regarding whole-body ICG was that the present study conducted both measurement methods sequentially, but not simultaneously. In the present study, the whole-body ICG measurement was performed either before RHC or on the following day at the latest, which may have contributed to the greater variance in precision of the whole-body ICG measurement compared with the RHC measurement. This variance was particularly noticeable for TPR and PVR and could be attributed to interim changes in the included parameters for calculation, e.g., blood pressure levels.

In summary, whole-body ICG measured the CO and CI of hospitalized CHF patients with acceptable accuracy. The measured TPR and PVR values also significantly correlated with the data derived from invasive RHC, but the accuracy of the measurement was limited. The results, therefore, raise the question of the extent to which whole-body ICG measurement can represent a reliable alternative to RHC measurement for TPR and PVR. Based on the available data, it is not possible to provide a conclusive answer to this question, so this should be investigated in future prospective multicenter studies. The feasibility of whole-body ICG is also limited by its own exclusion criteria, as patients with severe aortic or mitral valve regurgitation and severe aortic valve stenosis are not eligible for assessment. Nonetheless, these patients represent a considerable proportion of clinical care and will, therefore, continue to require RHC for measuring hemodynamic parameters. The extent to which valvular heart disease of moderate severity may have affected the accuracy of whole-body ICG measurement cannot be adequately evaluated from the available data and should, therefore, be investigated further in future studies. However, a relevant influence on the results by high-grade tricuspid valve regurgitation appears to be unlikely.

In the end, our study has demonstrated that whole-body ICG may be considered as an alternative measurement method for CO and CI in “real-world” hospitalized CHF patients with the right amount of caution and if the inclusion criteria are fulfilled.

## 5. Limitations

The present study had several limitations that should be considered when the results are interpreted.

A major limitation was encountered in the sequential but non-simultaneous measurement of the hemodynamic parameters between ICG and RHC measurements, which may have affected the accuracy of the whole-body ICG due to interim changes in the parameters. The accuracy of the measurements could also have been affected in patients with AF by the consecutive variation in ejection volumes, even if this was attempted to be compensated for by repeated measurements with subsequent averaging of the values. Besides the whole-body ICG measurement, further computations were necessary, e.g., echocardiographic measurement of PASP or the estimation of PCWP. The previously described limitations of these methods may also have affected the final accuracy of the whole-body ICG measurement. Owing to the retrospective design, no conclusions can be drawn with regard to possible causalities. The generalizability of the results may be impaired by the single-center design and the existing differences in clinical cohort characteristics between the present study cohort and the current multicenter CHF cohort.

## 6. Conclusions

Whole-body ICG can be considered an alternative measurement modality for CO and CI in hospitalized CHF patients if they meet the inclusion criteria for measurement. Further prospective studies are needed to clarify the correlation of NI-TPR and NI-PVR with invasive measurements.

## Figures and Tables

**Table 1:** Clinical cohort characteristics of patients undergoing right heart catheterization and whole-body ICG measurement.

Variable	Overall Cohort (*n* = 203)
Age (years)	72 ± 13
Male sex	65% (132)
BMI (kg/m^2^)	27 ± 5
Arterial hypertension	83.3% (169)
Coronary artery disease *Prior myocardial infarction* *Prior PCI* *Prior CAB-OP* *Prior CAB-OP + Valve replacement/repair*	49.3% (100) *19.7% (40)* *34.5% (70)* *11.3% (23)* *2% (4)*
Structural heart disease *Dilated cardiomyopathy* *Ischemic cardiomyopathy* *Hypertrophic cardiomyopathy* *Hypertrophic-obstructive cardiomyopathy* *Myocarditis* *Cardiac amyloidosis* *Cardiac sarcoidosis* *Pericarditis constrictiva* *Tachymyopathy*	85.7% (174) *17.2% (35)* *37.9% (77)* *3.9% (8)* *1% (2)* *8.9% (18)* *2% (4)* *1% (2)* *1% (2)* *12.8% (26)*
HFpEF HFmrEF HFrEF	53.7% (109) 12.3% (25) 34% (69)
Atrial fibrillation *Paroxysmal* *Persistent* *Permanent* *AF at time of baseline examination*	57.1% (116) *19.2% (39)* *26.1% (53)* *11.8% (24)* *29.1% (59)*
Diabetes mellitus *Oral antidiabetics* *Insulin*	23.2% (47) *16.3% (33)* *6.9% (14)*
Current smoker Former smoker	12.8% (26) 17.2% (35)
Hyperlipoproteinemia	57.1% (116)
NYHA I NYHA II NYHA III NYHA IV	13.8% (28) 42.4% (86) 40.4% (82) 3.4% (7)
NTproBNP (pg/mL) *	1271 ± 2192
GFR (mL/min)	63 ± 27
Urea (mg/dL)	50 ± 26
LDL-Cholesterol (mg/dL)	94 ± 39
HDL-Cholesterol (mg/dL)	57 ± 19
**Heart failure medication**	
ACE/AT1-inhibitors	65.5% (133)
Beta Blockers	82.8% (168)
ARNI	41.4% (84)
MRA	52.7% (107)
SGLT-II-inhibitors	69% (140)
Diuretics	75.9% (154)

* Median ± IQR. ACE—angiotensin-converting enzyme, AF—atrial fibrillation, ARNI—angiotensin-receptor–neprilysin-inhibitor, AT1—angiotensin receptor 1, BMI—body mass index, CAB-OP—coronary artery bypass operation, GFR—glomerular filtration rate, HDL—high-density lipoprotein, HFmrEF—heart failure with mildly reduced ejection fraction, HFpEF—heart failure with preserved ejection fraction, HFrEF—heart failure with reduced ejection fraction, ICG—impedance cardiography, LDL—low-density lipoprotein, MRA—mineralocorticoid receptor antagonist, NYHA—New York Heart Association, PCI—percutaneous coronary intervention.

**Table 2:** Echocardiographic characteristics of patients undergoing right heart catheterization and whole-body ICG measurement.

Variable	Overall Cohort (*n* = 203)
LVEDD (mm)	52 ± 11
LVESD (mm)	39 ± 12
Prior isolated surgical valve replacement/repair	25.1% (51)
Aortic valve regurgitation *None* *Mild* *Moderate* *Severe*	*60% (122)* *28.6% (58)* *11.3% (23)* *0% (0)*
Aortic valve stenosis *None* *Mild* *Moderate* *Severe*	*88.7% (180)* *5.4% (11)* *5.9% (12)* *0% (0)*
Mitral valve regurgitation *None* *Mild* *Moderate* *Severe*	*21.2% (43)* *55.7% (113)* *23.2% (47)* *0% (0)*
Prior M-TEER	12.3% (25)
Tricuspid valve regurgitation *None* *Mild* *Moderate* *Severe*	*18.2% (37)* *50.7% (103)* *15.3% (31)* *15.8% (32)*
Prior T-TEER *Cardioband*	1.5% (3) *1% (2)*

ICG—impedance cardiography, LVEDD—left ventricular end-diastolic diameter, LVESD—left ventricular end-systolic diameter, M-TEER—transcatheter edge-to-edge mitral valve repair, T-TEER—transcatheter edge-to-edge tricuspid valve repair.

**Table 3:** Hemodynamic measurements of whole-body ICG and right heart catheterization.

Variable	Overall Cohort (*n* = 203)
**Whole-body ICG**	
Heart rate (bpm)	72 ± 14
Systolic arterial pressure (mmHg) Mean arterial pressure (mmHg) Diastolic arterial pressure (mmHg)	131 ± 24 94 ± 15 76 ± 13
Stroke volume (mL)	58 ± 16
Stroke volume index (mL/m^2^)	30 ± 8
Cardiac output (L/min)	4.1 ± 1.3
Cardiac index (L/min/m^2^)	2.1 ± 0.6
Cardiac power index (W/m^2^)	0.45 ± 0.15
Total peripheral resistance (dyn × s^−1^ × cm^−5^)	2225 ± 3314
Total peripheral resistance index (dyn × s^−1^ × cm^−5^ × m^−^^2^)	3795 ± 1273
Pulmonary vascular resistance (dyn × s^−1^ × cm^−5^)	386 ± 218
Respiration rate (min^−1^)	17 ± 4
Total body water (kg) Proportion of total body water to weight (%)	44 ± 9 55 ± 10
Basal Impedance (Ω)	358 ± 60
**Right heart catheterization**	
Systolic pulmonary artery pressure (mmHg) Mean pulmonary artery pressure (mmHg) Diastolic pulmonary artery pressure (mmHg)	38 ± 16 24 ± 11 14 ± 8
Systolic right ventricular pressure (mmHg) Diastolic right ventricular pressure (mmHg)	38 ± 18 4 ± 4
Mean right atrial pressure (mmHg) *	6 ± 5
Mean pulmonary capillary wedge pressure (mmHg)	14 ± 8
Cardiac output (L/min)	4 ± 1.2
Cardiac index (L/min/m^2^)	2.1 ± 0.6
Total peripheral resistance (dyn × s^−1^ × cm^−5^)	1733 ± 590
Pulmonary vascular resistance (dyn × s^−1^ × cm^−5^)	220 ± 162
Pulmonal arterial saturation (%)	65 ± 9

* Median ± IQR, ICG—impedance cardiography.

## Data Availability

The raw data of this study are available upon request by contacting Felix Ausbuettel (felix.ausbuettel@staff.uni-marburg.de).

## Supplementary Materials

The following supporting information can be downloaded at: https://www.mdpi.com/article/10.3390/jcdd12040128/s1, Figure S1: Linear correlation between echocardiographic measurement of PASP + CVP and invasive measurement of PASP (A) and Bland-Altman plot comparing the mean difference ± 2 SD correlation between echocardiographic measurement of PASP + CVP and invasive measurement of PASP (B); Figure S2: Linear correlation analysis between RHC-CO and NI-CO (A), RHC-CI and NI-CI (B), RHC-TPR and NI-TPR (C) as well as RHC-PVR and NI-PVR (D) in patients without high-grade tricuspid valve regurgitation.

## References

[1] Roger V.L. (2013). Epidemiology of heart failure. Circ. Res..

[2] Jones N.R., Roalfe A.K., Adoki I., Hobbs F.D.R., Taylor C.J. (2019). Survival of patients with chronic heart failure in the community: A systematic review and meta-analysis. Eur. J. Heart Fail..

[3] McDonagh T.A., Metra M., Adamo M., Gardner R.S., Baumbach A., Böhm M., Burri H., Butler J., Čelutkienė J., Chioncel O. (2021). 2021 ESC Guidelines for the diagnosis and treatment of acute and chronic heart failure. Eur. Heart J..

[4] Fincke R., Hochman J.S., Lowe A.M., Menon V., Slater J.N., Webb J.G., LeJemtel T.H., Cotter G. (2004). Cardiac power is the strongest hemodynamic correlate of mortality in cardiogenic shock: A report from the SHOCK trial registry. J. Am. Coll. Cardiol..

[5] Paredes O.L., Shite J., Shinke T., Watanabe S., Otake H., Matsumoto D., Imuro Y., Ogasawara D., Sawada T., Yokoyama M. (2006). Impedance cardiography for cardiac output estimation: Reliability of wrist-to-ankle electrode configuration. Circ. J..

[6] Cotter G., Moshkovitz Y., Kaluski E., Cohen A.J., Miller H., Goor D., Vered Z. (2004). Accurate, noninvasive continuous monitoring of cardiac output by whole-body electrical bioimpedance. Chest.

[7] Kööbi T., Kaukinen S., Turjanmaa V.M., Uusitalo A.J. (1997). Whole-body impedance cardiography in the measurement of cardiac output. Crit. Care Med..

[8] Kööbi T., Kaukinen S., Turjanmaa V.M. (1999). Cardiac output can be reliably measured noninvasively after coronary artery bypass grafting operation. Crit. Care Med..

[9] Guha A., Arora D., Mehta Y. (2022). Comparative study of cardiac output measurement by regional impedance cardiography and thermodilution method in patients undergoing off pump coronary artery bypass graft surgery. Ann. Card. Anaesth..

[10] Kaukinen S., Kööbi T., Bi Y., Turjanmaa V.M.h. (2003). Cardiac output measurement after coronary artery bypass grafting using bolus thermodilution, continuous thermodilution, and whole-body impedance cardiography. J. Cardiothorac. Vasc. Anesth..

[11] Argueta E.E., Paniagua D. (2019). Thermodilution Cardiac Output: A Concept Over 250 Years in the Making. Cardiol. Rev..

[12] Kubiak G.M., Ciarka A., Biniecka M., Ceranowicz P. (2019). Right Heart Catheterization-Background, Physiological Basics, and Clinical Implications. J. Clin. Med..

[13] Tamimi O., Mohammed M.H.A. (2021). Pulmonary Vascular Resistance Measurement Remains Keystone in Congenital Heart Disease Management. Front. Cardiovasc. Med..

[14] Hill L.K., Sollers Iii J.J., Thayer J.F. (2013). Resistance reconstructed estimation of total peripheral resistance from computa-tionally derived cardiac output. Biomed. Sci. Instrum..

[15] Chemla D., Castelain V., Humbert M., Hébert J.-L., Simonneau G., Lecarpentier Y., Hervé P. (2004). New formula for pre-dicting mean pulmonary artery pressure using systolic pulmonary artery pressure. Chest.

[16] Chioncel O., Lainscak M., Seferovic P.M., Anker S.D., Crespo-Leiro M.G., Harjola V.-P., Parissis J., Laroche C., Piepoli M.F., Fonseca C. (2017). Epidemiology and one-year outcomes in patients with chronic heart failure and preserved, mid-range and reduced ejection fraction: An analysis of the ESC Heart Failure Long-Term Registry. Eur. J. Heart Fail..

[17] Joseph P., Dokainish H., McCready T., Budaj A., Roy A., Ertl G., Gomez-Mesa J.E., Leong D., Ezekowitz J., Hage C. (2020). A multinational registry to study the characteristics and outcomes of heart failure patients: The global congestive heart failure (G-CHF) registry. Am. Heart J..

[18] Imhoff M., Lehner J.H., Löhlein D. (2000). Noninvasive whole-body electrical bioimpedance cardiac output and invasive thermodilution cardiac output in high-risk surgical patients. Crit. Care Med..

[19] Kööbi T., Kaukinen S., Ahola T., Turjanmaa V.M. (1997). Non-invasive measurement of cardiac output: Whole-body impedance cardiography in simultaneous comparison with thermodilution and direct oxygen Fick methods. Intensive Care Med..

[20] Bhavya G., Nagaraja P.S., Singh N.G., Ragavendran S., Sathish N., Manjunath N., Kumar K.A., Nayak V.B. (2020). Com-parison of continuous cardiac output monitoring derived from regional impedance cardiography with continuous ther-modilution technique in cardiac surgical patients. Ann. Card. Anaesth..

[21] Taniguchi Y., Emoto N., Miyagawa K., Nakayama K., Kinutani H., Tanaka H., Shinke T., Hirata K. (2013). Noninvasive and simple assessment of cardiac output and pulmonary vascular resistance with whole-body impedance cardiography is useful for monitoring patients with pulmonary hypertension. Circ. J..

[22] Martelli G., Congedi S., Lorenzoni G., Nardelli M., Lucchetta V., Gregori D., Tiberio I. (2023). Echocardiographic assessment of pulmonary capillary wedge pressure by E/e’ ratio: A systematic review and meta-analysis. J. Crit. Care.

