# Supplementary material for: Non-Invasive Measurement of Hemodynamic Parameters via Whole-Body Impedance Cardiography Among Hospitalized Heart Failure Patients: An Effective Alternative to Invasive Right Heart Catheterization?
Source: J Cardiovasc Dev Dis. 2025 Apr 2;12(4):128. doi: 10.3390/jcdd12040128 (PMC12027845; doi:10.3390/jcdd12040128)
Supplement: Supplementary file 1 [file jcdd-12-00128-s001.zip › jcdd-3486037-supplementary.pdf]

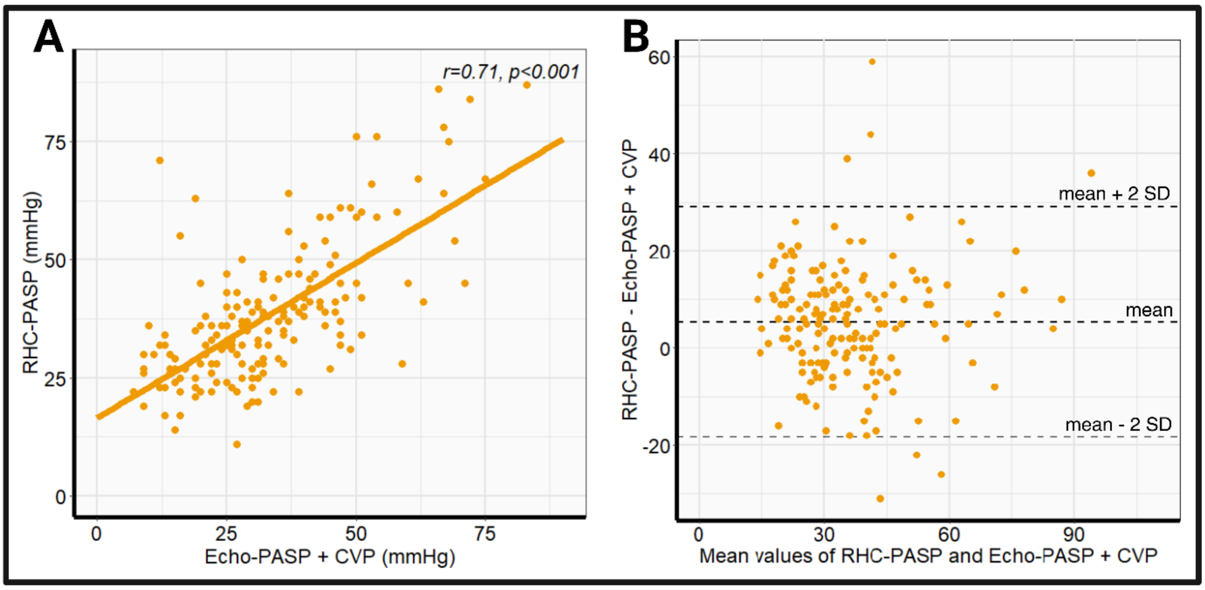


**Figure S1: Linear correlation between echocardiographic measurement of PASP + CVP and invasive measurement of PASP (A) and Bland-Altman plot comparing the mean difference ± 2 SD correlation between echocardiographic measurement of PASP + CVP and invasive measurement of PASP (B)**

*CVP – central venous pressure, Echo-PASP – echocardiographically-measured pulmonary artery systolic pressure, RHC-PASP – Right Heart Catheter-derived pulmonary artery systolic pressure, SD – standard deviation*

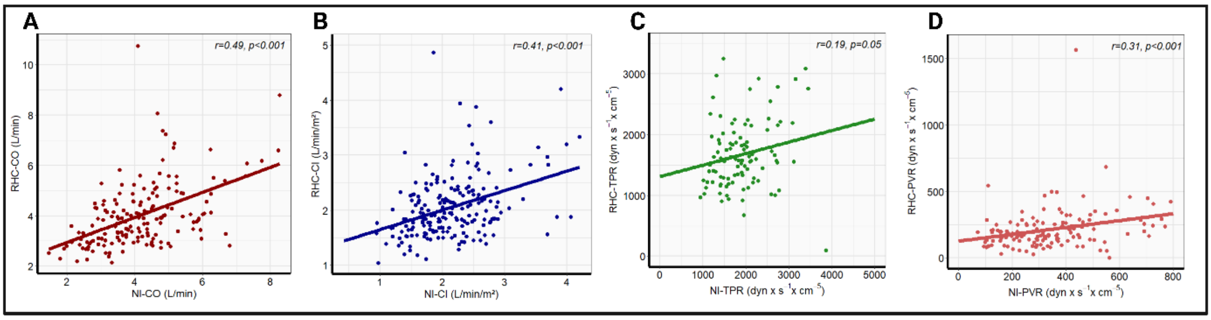


**Figure S2. Linear correlation analysis between RHC-CO and NI-CO (A), RHC-CI and NI-CI (B), RHC-TPR and NI-TPR (C) as well as RHC-PVR and NI-PVR (D) in patients without high-grade tricuspid valve regurgitation.**

*NI-CI – non-invasively measured Cardiac index, NI-CO – non-invasively measured Cardiac output, NI-PVR – non-invasively measured pulmonary vascular resistance, NI-TPR – non-invasively measured total peripheral resistance, RHC-CI – Right Heart Catheter-derived Cardiac output, RHC-CO – Right Heart Cathe-ter-derived Cardiac index, RHC-PVR – Right Heart Catheter-derived pulmonary vascular resistance, RHC-TPR – Right Heart Catheter-derived total peripheral resistance.*
